# Abderhalden Test

**Published:** 1914-06

**Authors:** 


**Abderhalden Test.** L. Michaelis and L. von Langermarck, Deutsche Med. Woch., Feb. 12, 1914, deny that the serum of pregnant women contains a specific ferment differing from that of the non-pregnant or even of man.

